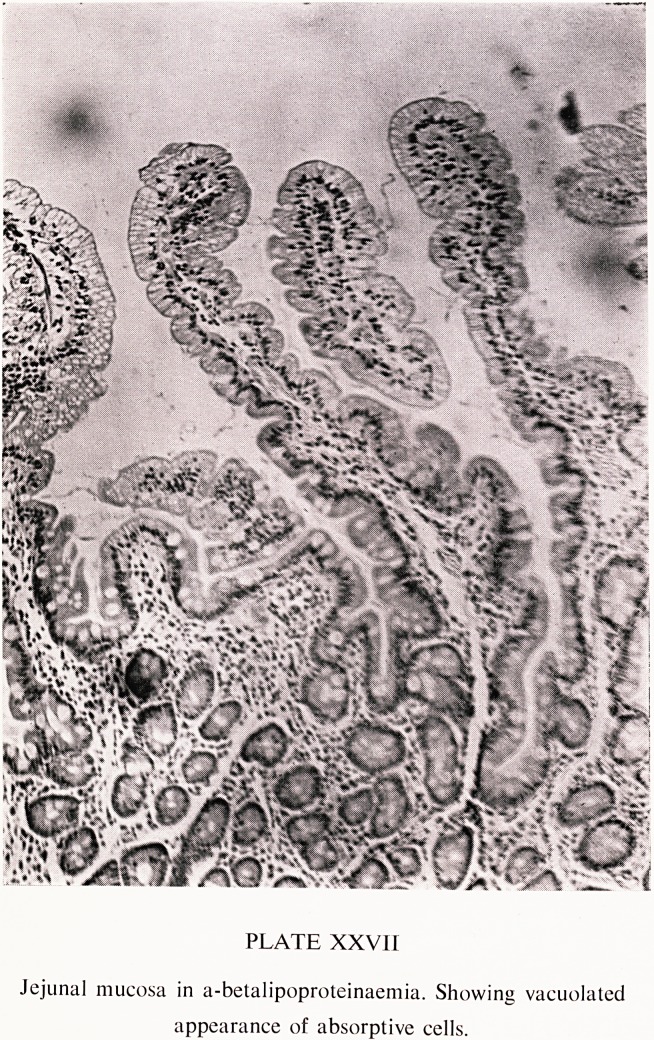# Lipoprotein Deficiency Disorders

**Published:** 1969-10

**Authors:** June K. Lloyd


### LIPOPROTEIN DEFICIENCY DISORDERS

June K. Lloyd

Hypolipidaemia may occur in a number of conditions, and Table I gives the most important causes, together with the lipoprotein species primarily affected. In this paper, only the two genetically determined primary disorders, abetalipoproteinaemia and familial alphalipoprotein deficiency (Tangier disease), will be considered. The study of the clinical and pathological manifestations of these diseases has been of prime importance in enlarging our concepts of the normal functions of the two major serum lipoproteins.

TABLE I

**Hypolipidaemia**

| Lipoprotein | Primary | Secondary |
|---|---|---|
| Chylomicron | | Malabsorption<br>A-beta-lipoproteinaemia |
| beta-Lipoprotein | A-beta-lipoproteinaemia<br>(physiological at birth) | Malabsorption<br>Hyperthyroidism<br>Chronic anaemia<br>Liver failure |
| alpha-Lipoprotein | Tangier disease | Pre-beta-lipoproteinaemia<br>Liver disease<br>Non-specific |

### ABETALIPOPROTEINAEMIA

This rare disease, which has now been described in about 35 patients, was first recognised in 1950 by Bassen and Kornzweig, who reported two patients with neurological abnormalities and abnormally shaped erythrocytes. The latter were named acanthocytes (*acanthos*, a spine or thorn in Greek) after the description by Singer *et al.* (1952). Hypocholesterolaemia was first observed by Jampel and Falls in 1958, and in 1960 three groups of workers independently showed that betalipoprotein was absent from the blood (Lamy *et al.;* Mabry *et al.;* Salt *et al.*). The primary gene effect was orginally thought to concern the synthesis of the protein moiety of betalipoprotein, but recent work by Lees (1967) has shown that the B-protein is present in the sera of patients and suggests that the basic abnormality concerns the formation of the complete lipoprotein macromolecule. The condition is inherited as an autosomal recessive; the heterozygotes have no clinical or haematological abnormalities, and in only one set of parents have low levels of betalipoprotein been found (Salt *et al.*, 1960).

CLINICAL AND LABORATORY FEATURES

The main features of the disorder are acanthocytosis and steatorrhoea which are present from birth, and an ataxic neuropathy and a pigmentary retinopathy which develop in later childhood or adolescence and are slowly progressive.

*The lipoprotein abnormality.* Absence of betalipoprotein from the serum can be demonstrated by paper electrophoresis, by immunochemical techniques, and by ultracentrifugation. The latter technique also shows that alphalipoprotein is reduced to between one half to one third of its normal concentration (Jones and Ways, 1967). The serum lipids reflect the lipoprotein changes; total cholesterol, phospholipid, and triglyceride concentrations are very low, cholesterol levels usually being of the order of 20-40 mg./100 ml. Estimation of the individual phospholipids shows a relative increase in sphingomyelin and a decrease in phosphatidyl choline, and levels of linoleic acid in the latter fraction are greatly reduced. Vitamin E and carotenoid pigments which are transported by betalipoprotein are absent from the serum, and vitamin A levels are low.

*The red cell abnormality.* Acanthocytosis is always present, and is best seen in a fresh wet undiluted preparation (Plate XXVI); the appearances on a dried film have been mistaken for crenation. Normal rouleaux formation is absent and the sedimentation rate is therefore abnormally low. Osmotic fragility is normal or only slightly increased. Red cell survival time is slightly shortened but clinically significant intravascular haemolysis is not a feature However, autohaemolysis and peroxidative haemolysis are considerably increased and this abnormality can be corrected both *in vitro* by alphatocopherol acetate, and after administration of this vitamin to the patient. Studies of red cell membrane lipids show normal concentrations of total cholesterol and total phospholipid, but the distribution of the individual phospholipids is abnormal with an increase in sphingomyelin and decrease in phosphatidyl choline. It is probable that this abnormality is secondary to the phospholipid abnormality in the serum (Ways and Simon, 1964).

*The malabsorptive defect.* Symptoms due to steatorrhoea are usually the presenting feature of the disease in childhood. Duodenal concentrations of pancreatic enzymes and bile acids are normal and intestinal biopsy shows normal villous architecture. The mucosal cells, however, are vacuolated and foamy in appearance (Plate XXVII) and histochemical staining shows a great accumulation of lipid which on chemical analysis has been found to be largely triglyceride (Ways *et al.*, 1967). The lipid accumulation is due to failure of chylomicron formation; the reason for this is not clear but possibly the enzyme system responsible for betalipoprotein synthesis is also necessary for chylomicron production Although the chylomicron route of fat absorption is completely blocked, the degree of steatorrhoea is not gross, about 80% of fat being absorbed. The absorbed fatty acids are presumably transported by the portal blood stream (Kayden and Medick, 1967).

*The neurological abnormality.* Clinical evidence of the neurological and retinal lesions appears towards the end of the first decade. The neurological mani-

PLATE XXVI

Acanthocytes in a-betalipoproteinaemia. Stained dried blood film (bottom) and fresh wet preparation of whole blood (top).

Reproduced from the Archives of Disease in Childhood, 1968, 44, 398 by permission of the editor and publishers.

PLATE XXVII

Jejunal mucosa in a-betalipoproteinaemia. Showing vacuolated appearance of absorptive cells.

festations resemble those of Friedreich's ataxia and the disability is slowly progressive. The retinopathy is characterized by pigmentary degeneration which involves both the periphery and the macula The pathogenesis of the lesions is not understood. The possibilities include abnormalities of lipid composition of neurological and retinal tissue secondary to the primary lipoprotein disturbance, or the effects of long term deficiencies of linoleic acid, of vitamin A, or of vitamin E. To date no detailed studies of brain or retinal lipids have been made. Linoleic acid deficiency appears to be a consequence of the malabsorption, and similar low levels have been recorded in other disorders. Vitamin A deficiency is unlikely to be the sole explanation as no other signs of vitamin A deficiency are present, and the maintenance of normal serum levels (by giving large doses of a water-miscible preparation) from the age of 17 months did not prevent the appearance of retinopathy at the age of 5 years in one of our patients (Wolff *et al.* 1964). Vitamin E deficiency may be a contributory factor as no deterioration has occurred in our most severely affected patient during a three year period of treatment with this vitamin (Lloyd, Harries and Muller, unpublished).

### DIAGNOSIS

The diagnosis should be suspected in all cases of steatorrhoea, of retinitis pigmentosa, or of a Friedreich-like ataxia. A serum cholesterol level below 60 mg/100 ml. together with acanthocytosis of the red cells is virtually diagnostic; confirmation of the lipoprotein abnormality can then be obtained by electrophoretic, ultracentrifugal, or immunochemical studies. Acanthocytosis alone is not pathognomic as the red cell abnormality has been described in other diseases, notably severe liver disease. It has also become apparent that familial deficiency, as distinct from absence, of betalipoprotein occurs and may be associated with some, but not all, of the features of abetalipoproteinaemia.

### PROGNOSIS

Patients reaching adult life have usually been severely crippled by neurological disease. One adult patient died with cardiac arrhythmia (Sobrevilla *et al.* 1964), and one child has also died suddenly (D. S. Fredrickson, personal communication).

### TREATMENT

Steatorrhoea can be abolished by restriction of dietary fat, and on such a regime normal growth has been achieved in two of our patients (Lloyd, 1968). Because of the low levels of linoleic acid in the serum and red cell lipids, some of the dietary fat should be polyunsaturated. If adequate caloric intake cannot be achieved with a low-fat diet, medium chain triglycerides, the fatty acids of which are transported almost entirely by the portal vein, may be used (Leyland *et al.* 1969). Fat-soluble vitamins should be given in a water-miscible form. Very large doses of A (up to 20,000 i.u. daily) and E (1-2 g. daily) are required. It is of interest that rickets has only rarely been observed, and large doses of vitamin D are not needed.

### FAMILIAL ALPHALIPOPROTEIN DEFICIENCY

### (Tangier Disease)

The discovery of a primary alphalipoprotein deficiency disorder came in 1960 when a 5 year old boy from Tangier Island in Chesapeake Bay underwent the common paediatric operation of tonsillectomy. The large size and remarkable yellow-orange colouration of his tonsils provoked further investigations and virtual absence of alphalipoprotein was demonstrated in his serum. Similar findings were present in his sister, and the condition was named Tangier disease after their island home (Fredrickson *et al.*, 1961). Since this time a further eight patients have been described, including three pairs of siblings (Fredrickson, 1966; Kocen *et al.*, 1967; Engel *et al.*, 1967; Kummer *et al.*, 1968). The condition is inherited as an autosomal recessive, and heterozygotes have reduced concentrations of alphalipoprotein in the serum.

### CLINICAL AND LABORATORY FEATURES

The main features of the disorder are due to the deposition of cholesterol esters in many tissues of the body, particularly those of the reticuloendothelial system.

*The lipoprotein abnormality.* Normal alphalipoprotein is absent from the serum but small amounts of an immunochemically distinct alphalipoprotein (designated alpha T) have been demonstrated (Levy and Fredrickson, 1966). The serum is characteristically turbid in the fasting state because of moderate elevation of triglyceride concentrations (200-300 mg./100 ml.). Concentrations of total cholesterol are reduced to 50-100 mg./100 ml. and of phospholipids to 60-140 mg./100 ml. The distribution of the phospholipids shows an increase in the percentage of phosphatidyl choline and a decrease in sphingomyelin, which is the opposite of the abnormality found in abetalipoproteinaemia Ultracentrifugal separation of the lipoproteins shows an excess of very low-density lipoproteins (corresponding to pre-betalipoproteins on paper electrophoresis), and analysis of the fractions shows a relative increase of triglyceride in all fractions including the small amount of high-density (alpha) lipoprotein (Kocen *et al.*, 1967).

*Clinical and pathological findings.* The major abnormalities in the 10 patients so far described are summarized in Table II. The lipid accumulation in the tissues has been shown histochemically and by analysis to be predominately esterified cholesterol (Fredrickson, 1966). The most striking and pathognomonic accumulation occurs in the tonsils, and even if the latter have been removed the colouration of the remaining tags of tissue in the tonsillar fossae and of the pharyngeal lymph follicles is easily appreciated.

Malabsorption is not a feature, lipid accumulation does not occur in the small intestinal mucosa, and chylomicron production is normal, although the amount of cholesterol in the chylomicrons appears to be decreased (Fredrickson, 1966).

Red cell morphology is normal, but the phospholipid composition of the red cell membrane shows abnormalities similar to those found in the plasma (relative increase in phosphatidyl choline and decrease in sphingomyelin) and

TABLE II

**Clinical Features of Tangier Disease**

| Patients | Sex | Age | Tonsils | Liver Spleen | CNS | Cornea | Marrow | Rectal Mucosa |
|---|---|---|---|---|---|---|---|---|
| Tangier | M | 5 | + | + | | | + | |
| Tangier | F | 6 | + | | | | | |
| Missouri | F | 8 | + | | | | | |
| Missouri | F | 12 | + | + | | | | |
| Kentucky | M | 45 | + | | | + | + | + |
| Kentucky | M | 48* | + | | | + | + | + |
| London | M | 38 | + | + | + | (+) | + | + |
| New Orleans | F | 16 | + | + | + | | + | |
| New Orleans | F | 24 | | | + | + | | + |
| Bern | M | 40 | + | + | + | + | + | + |

* Died ? Cardiac Infarction the opposite of those found in acanthocytes in abetalipoproteinaemia (Shacklady *et al.*, 1968).

DIAGNOSIS

The diagnosis should be suspected in patients presenting with hepatosplenomegaly, unusual forms of neuropathy, or corneal opacities; and the appearances of the oropharynx are pathognomonic. The lipoprotein abnormality may be suspected by the finding of a raised serum triglyceride concentration combined with a low serum cholesterol concentration, and confirmed by electrophoretic, ultarcentrifugal, and immunochemical studies.

Very low concentrations of alphalipoprotein may also occur in association with severe liver disease, in patients with familial lecithin-cholesterol acyl transferase deficiency (Norum and Gjone, 1967), and in association with certain of the lipid storage diseases. The differential diagnosis does not usually present problems, but family studies should probably always be made to demonstrate the familial nature of the alphalipoprotein deficiency in Tangier disease.

### PROGNOSIS

One patient has died at the age of 48 years, probably from myocardial infarction. Peripheral neuropathy has proved moderately incapacitating to some patients. Four of the ten patients are still children and the ultimate prognosis is not known.

### TREATMENT

No specific treatment is available. The hypertriglyceridaemia has been shown to respond to reduction in the dietary carbohydrate intake (Levy *et al.*, 1966) and in one adult patient a low carbohydrate diet together with Clofibrate is currently being given (Lloyd *et al.*, unpublished).

### Conclusions

From the study of these two primary lipoprotein deficiency disorders certain conclusions can be drawn regarding the normal functions of beta- and alphalipoproteins.

1. Betalipoprotein performs an essential carrier function for carotenoids and vitamin E.
2. Betalipoprotein (or the enzyme systems necessary for its formation) appears to be essential for the movement of triglyceride out of cells and in particular the intestinal cells. Alphalipoprotein appears to be necessary for the transport of cholesterol ester out of cells; it is clearly not essential for chylomicron formation.
3. Both lipoproteins probably play a part in maintaining the normal lipid composition of cell membranes. This is demonstrable in the red cell membrane but may also affect other membranes.

The mechanisms by which the lipoproteins perform the various functions are still not understood.

### References

Bassen, F. A. and Kornzweig, A. L. (1950). Blood, *5*, 381.

Engel, W. K., Dorman, J. D., Levy, R. I. and Fredrickson, D. S. (1967). Archives of Neurology, *17*, 1.

Fredrickson, D. S., Altrocchi, P. H., Avioli, L. V., Goodman, De W. S. and Goodman, H. C. (1961). Annals of Internal Medicine, *55*, 1016.

Fredrickson, D. S. (1966) *in* The Metabolic Basis of Inherited Disease, ed. J. B. Stanbury, J. B. Wyngaarden and D. S. Fredrickson, p. 486.

Jampel, R. S. and Falls, H. F. (1958). American Medical Association Archives of Ophthalmology, *59*, 818.

Jones, J. W. and Ways, P. (1967). Journal of Clinical Investigation, *46*, 1151.

Kayden, H. J. and Medick, M. (1967). Journal of Clinical Investigation, *46*, 1077.

Kocen, R. S., Lloyd, J. K., Lascelles, P. T., Fosbrooke, A. S. and Williams, D. (1967). Lancet, *1*, 1341.

Kummer, H., Laissue, J., Spiess, H., Pflugshaupt, R. and Bucher, U. (1968). Schweizerische Medizinische Wochenschrift, *98*, 406.

Lamy, M., Frezal, J., Polonovski, J. and Rey, J. (1960). Comptes rendus des Séances de la Société de Biologie, *154*, 1974.

Lees, R. S. (1967). Journal of Lipid Research, *8*, 396.

Levy, R. I. and Fredrickson, D. S. (1966). Circulation, 33-34, Suppl. 3, 156.

Levy, R. I., Lees, R. S. and Fredrickson, D. S. (1966). Journal of Clinical Investigation, *45*, 63.

Leyland, F. C., Fosbrooke, A. S., Lloyd, J. K., Segall, M. M., Tamir, I., Tomkins, R. and Wolff, O. H. (1969). Archives of Disease in Childhood, *44*, 170.

Lloyd, J. K. (1968). Archives of Disease in Childhood, *43*, 393.

Mabry, C. C., Di George, A. M. and Auerbach, V. H. (1960). Clinical Research, *8*, 371.

Norum, K. R. and Gjone, E. (1967). Scandinavian Journal of Clinical and Laboratory Investigation, *20*, 231.

Salt, H. B., Wolff, O. H., Lloyd, J. K., Fosbrooke, A. S., Cameron, A. H. and Hubble, D. V. (1960). Lancet, *2*, 325.

Shacklady, M. M., Djardjouras, E. M. and Lloyd, J. K. (1968). Lancet, *2*, 151.

Singer, K., Fisher, B. and Perlstein, M. A. (1952). Blood, *7*, 577.

Sobrevilla, L. A., Goodman, M. L. and Kane, C. A. (1964). American Journal of Medicine, *37*, 821.

Ways, P. O. and Simon, E. R. (1964). Journal of Clinical Investigation, *43*, 1322.

Ways, P. O., Parmentier, C. M., Kayden, H. J., Jones, J. W., Saunders, D. R. and Rubin, C. E. (1967). Journal of Clinical Investigation, *46*, 35.

## Figures and Tables

**PLATE XXVI f1:**
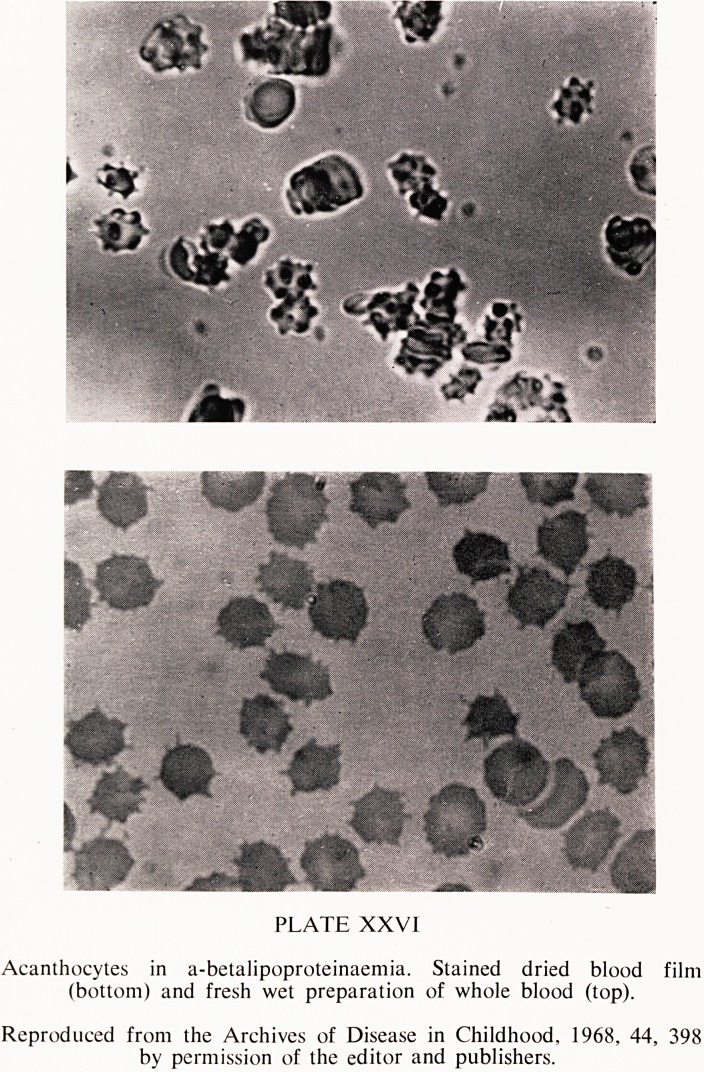


**PLATE XXVII f2:**